# Associations between TGF-*β*1 Levels and Markers of Hemolysis, Inflammation, and Tissue Remodeling in Pediatric Sickle Cell Patients

**DOI:** 10.1155/2021/4651891

**Published:** 2021-03-13

**Authors:** Rayra P. Santiago, Magda O. S. Carvalho, Camylla V. B. Figueiredo, Luciana M. Fiuza, Rodrigo M. Oliveira, Sètondji C. M. A. Yahouédéhou, Valma M. L. Nascimento, Isa M. Lyra, Théo Araujo-Santos, Nívea F. Luz, Milena M. Aleluia, Caroline C. Guarda, Valéria M. Borges, Marilda S. Goncalves

**Affiliations:** ^1^Instituto Gonçalo Moniz, Fundação Oswaldo Cruz, 40.296-710 Salvador, Brazil; ^2^Universidade Federal da Bahia, 40.170-110 Salvador, Brazil; ^3^Hospital Universitário Professor Edgard Santos, Universidade Federal da Bahia, 40110-060 Salvador, Brazil; ^4^Fundação de Hematologia e Hemoterapia do Estado da Bahia, 40286-240 Salvador, Brazil; ^5^Departamento de Ciências Biológicas, Universidade Estadual de Santa Cruz, 45.662-900 Ilhéus, Brazil

## Abstract

Transforming growth factor beta (TGF-*β*) is a cytokine with important involvement in biological processes related to the pathogenesis of sickle cell disease (SCD), including endothelial and vascular dysfunction, inflammation, and hematopoietic homeostasis. This study is aimed at investigating associations between levels of TGF-*β*1 and classical laboratory biomarkers and inflammatory mediators, as well as the tissue inhibitor of metalloproteases-1 (TIMP-1) and matrix metalloproteinase-9 (MMP-9), in pediatric patients (*n* = 123) with SCD in steady state: 84 with sickle cell anemia (HbSS) and 39 with hemoglobin SC disease (HbSC). A healthy control (HC) group of 59 individuals was also included. Hematological and biochemical analyses were carried out using electronic methods. TGF-*β*1, TIMP-1, and MMP-9 plasma quantifications were performed by ELISA. TGF-*β*1 plasma levels were higher in HbSS individuals than in HbSC and HC. In individuals with HbSS, TGF-*β*1 levels were positively correlated with red blood cells, hemoglobin, hematocrit, platelets, and TIMP-1. In addition, HbSS individuals with TGF-*β*1 levels above the median (≥72.29 ng/mL) also presented increased monocyte counts and decreased albumin levels. In patients with HbSC, TGF-*β*1 levels were positively correlated with leukocytes, eosinophils, lymphocytes, monocytes, and platelets, as well as levels of TIMP-1, VLDL-C, triglycerides, heme, and AST. Additionally, HbSC individuals with TGF-*β*1 levels above the median (≥47.80 ng/mL) presented increased leukocyte and platelet counts, as well as increased levels of triglycerides, VLDL-C, MMP-9, and TIMP-1, and decreased HDL-C. Our findings suggest that TGF-*β*1 may play important roles in vascular remodeling, vasculopathy, angiogenesis, and inflammation in pediatric patients with SCD.

## 1. Introduction

Sickle cell disease (SCD) is a group of disorders characterized by the presence of the hemoglobin variant S (HbS). Sickle cell anemia (SCA), the most severe type of SCD, represents homozygosity in the inheritance of the beta allele S (HbSS). Hemoglobin SC disease (HbSC) is characterized by the association of HbS with another hemoglobin variant, hemoglobin C (HbC), and is considered a milder phenotype of SCD [1–4].

Individuals with SCD exhibit an acute and chronic inflammatory status associated with recurrent infections and increased leukocyte counts, as well as the activation of leukocytes, red blood cells, reticulocytes, and endothelial cells. These individuals often experience clinical events related to vascular dysfunction, such as priapism, pulmonary hypertension, vasoocclusive crisis (VOC), and stroke [5, 6].

Vascular dysfunction is a common feature in SCD, mainly caused by nitric oxide (NO) imbalance in association with chronic hemolysis [5, 7]. During intravascular hemolysis, free hemoglobin and heme react with NO, causing the degradation of this free radical. The hemolytic process also induces the release of arginase, which consumes arginine, the main substrate for NO production. These events result in reactive oxygen and nitrogen species (ROS and RNS) production, which contribute to cell damage and vascular dysfunction by way of nitrosative stress [6].

The chronic inflammatory status observed in SCD has also been linked to increased levels of inflammatory cytokines, such as interleukin- (IL-) 1, tumor necrosis factor-alpha (TNF-*α*), and endothelin-1, which are produced by activated endothelial cells [8]. SCA patients have been described to present increased plasma levels of TNF-*α*, IL-8, and prostaglandin E_2_ (PGE_2_) [9]. In addition, transforming growth factor beta 1 (TGF-*β*1), IL-17, and IL-18 are capable of activating the vascular endothelium [8, 10]. In SCD, patterns of cytokine production vary during steady and crisis state [11], suggesting that the inflammatory response is capable of modulating clinical events.

TGF-*β* is a pleiotropic family of cytokines produced by many cell types, such as immune cells (T cells and macrophages), tumor cells, and stromal cells. These growth factors have been implicated in the regulation of cell growth, proliferation, differentiation, adhesion, and migration, as well as Th17 response polarization, ROS production, and apoptosis [12–16]. In the context of SCD, TGF-*β* is involved in several processes, including wound healing/ulceration, proliferative vasculopathy, inflammation, immune response, and ROS production [10, 17], all of which have been linked to clinical events.

TGF-*β* can upregulate the expression and activity of matrix metalloproteinases 9 (MMP-9), as well as its antagonist, tissue inhibitor of metalloproteases-1 (TIMP-1) [18, 19]. MMP-9, a zinc-dependent endopeptidase that acts in the extracellular matrix, promotes tissue remodeling in response to physiological and pathological conditions [18–21] Additionally, MMP-9 can also activate cytokine and chemokine production and is a regulator of inflammation and immunity [10]. Interplay between TIMP-1 and MMP-9 is involved in angiogenesis, cell growth, and apoptosis [22, 23].

Considering the immunomodulatory role of TGF-*β* together with the plethora of effects exerted by this superfamily, we hypothesized that TGF-*β*1 may play a key role in the pathogenesis of SCD. Hence, we sought to investigate associations between plasma levels of TGF-*β*1 and classical laboratory biomarkers, as well as TIMP-1 and MMP-9, in individuals with HbSS and HbSC.

## 2. Material and Methods

### 2.1. Subjects

Pediatric patients with SCD (*n* = 123) were recruited from the Bahia State Hematology and Hemotherapy Foundation (HEMOBA), located in Salvador, Brazil. Of these, 84 were HbSS and 39 were HbSC; 37 (44.04%) and 19 (48.71%) were female, respectively. All individuals were in steady-state, signifying the absence of acute events in the three months prior to inclusion, and none were undergoing hydroxyurea therapy. The mean age of patients with HbSS and HbSC was 8.76 ± 3.78 and 10.72 ± 4.24 years, respectively.

A healthy control (HC) group consisting of 59 individuals was included, 30 (50.85%) of whom were female, with mean age of 8.38 ± 3.50 years. These individuals were recruited from the Laboratory of Clinical and Toxicological Analysis, College of Pharmaceutical Sciences, Federal University of Bahia (LACTFAR-UFBA).

This research protocol was approved by the institutional research board of the Gonçalo Moniz Institute (protocol number: 0016.0.225.000-09) and was conducted in accordance with the 1964 Declaration of Helsinki and its subsequent revisions. All individuals were informed regarding the purpose and procedures of this study, and informed written consent was obtained from each patient's legal guardian.

### 2.2. Hematological and Biochemical Parameters

Hematological parameters were quantified using a Coulter Count T-890 electronic cell counter (Coulter Corporation, Hialeah, Florida, USA). Reticulocytes were counted after staining supravitally with brilliant cresyl blue dye. Hemoglobin profiles and fetal hemoglobin (HbF) levels were determined by high-performance liquid chromatography using an HPLC/Variant-II hemoglobin testing system (Bio-Rad, Hercules, California, USA).

Biochemical parameters were measured using an automated A25 chemistry analyzer (Biosystems S.A., Barcelona, Catalunya, Spain). Serum ferritin was measured by immunoassay using an Access® 2 immunoassay system (Beckman Coulter Inc., Pasadena, California, USA). In addition, alpha 1 antitrypsin (AAT) levels were determined using an IMMAGE® Immunochemistry System (Beckman Coulter Inc., Pasadena, California, USA). Total systemic free heme was measured in plasma samples using the QuantiChrom Heme Assay Kit (BioAssay Systems, Hayward, California, USA) following the manufacturer's protocol.

Laboratory analysis was performed at the Laboratory of Genetic Investigation and Translational Hematology at the Gonçalo Moniz Institute-FIOCRUZ (LIGHT-IGM/FIOCRUZ) and at LACTFAR-UFBA.

### 2.3. TGF-*β*1, TIMP-1, and MMP-9 Plasma Measurement

TGF-*β*1, TIMP-1, and MMP-9 plasma levels were measured by ELISA (R&D Systems, Minneapolis, Minnesota, USA) in accordance with the manufacturer's protocol.

### 2.4. Statistical Analysis

All analyses were performed using the Statistical Package for the Social Sciences (SPSS) software, version 20.0 (IBM, Armonk, New York, USA) and GraphPad Prism version 6.0 (GraphPad Software, San Diego, California, USA), which was also used for graph assembly. Significance was considered when *p* < 0.05. Variable values were summarized as means. To perform comparisons among SCD phenotypes, subgroups of individuals were formed according to TGF-*β*1 median values. The Shapiro-Wilk test was used to determine the distribution of quantitative variables. Depending on distribution, comparisons of two numerical variables were performed using the independent *t*-test and Mann–Whitney *U* test. Ordinary one-way ANOVA or Kruskal-Wallis test was performed to compare three numerical variables depending on distribution. Spearman's rank correlation coefficient or Pearson's correlation coefficient were used to measure the strength of linear relationships between paired variables.

## 3. Results

### 3.1. Hematological and Biochemical Laboratory Parameters

Table 1 lists the hematological and biochemical laboratory parameters of the investigated HbSS, HbSC, and HC individuals. Comparisons among the hematological and biochemical parameters in these individuals revealed statistically significant differences in hemolytic and inflammatory markers, leukocyte counts, as well as biomarkers of lipid and iron metabolism, and hepatic and renal function.

In comparison to individuals with HbSC and HC, patients with HbSS presented significantly decreased red blood cell (RBC) counts and hemoglobin (Hb), hematocrit (Ht), high-density lipoprotein cholesterol (HDL-C), urea, and creatinine levels, in addition to increased mean corpuscular volume (MCV), mean corpuscular hemoglobin (MCH), mean corpuscular hemoglobin concentration (MCHC), total bilirubin, indirect bilirubin, direct bilirubin, lactate dehydrogenase (LDH), fetal hemoglobin (HbF), iron, very low-density lipoprotein cholesterol (VLDL-C), triglyceride, aspartate aminotransferase (AST), alanine aminotransferase (ALT), alpha 1 antitrypsin (AAT) and ferritin levels, as well as counts of reticulocytes, leukocytes, neutrophils, eosinophils, lymphocytes, monocytes, and platelets.

### 3.2. HbSS Individuals Present Increased Plasma Levels of TGF-*β*1

**Figure 1:**
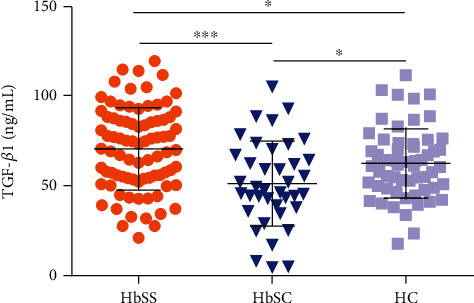
TGF-*β*1 plasmatic levels in individuals with HbSS, HbSC, and healthy controls (HC). ^∗^*p* < 0.05; ^∗∗∗^*p* < 0.0001.

HbSS individuals presented higher plasmatic TGF-*β*1 levels than HC and HbSC individuals (SS > HC > SC). Individuals with HbSS presented mean TGF-*β*1 levels of 70.80 ± 23.11 ng/mL, with median values of 72.29 ng/mL (IQR: 54.23–87.68 ng/mL), versus HC: mean 62.63 ± 19.44 ng/mL and median 61.90 ng/mL (IQR: 49.10–73.60 ng/mL). Individuals with HbSC presented mean TGF-*β*1 levels of 51.43 ± 23.76 ng/mL and a median value of 47.80 ng/mL (IQR: 38.24–67.51 ng/mL) (Figure 1).

### 3.3. Correlations between TGF-*β*1 Plasma Levels and Laboratory Parameters

**Figure 2:**
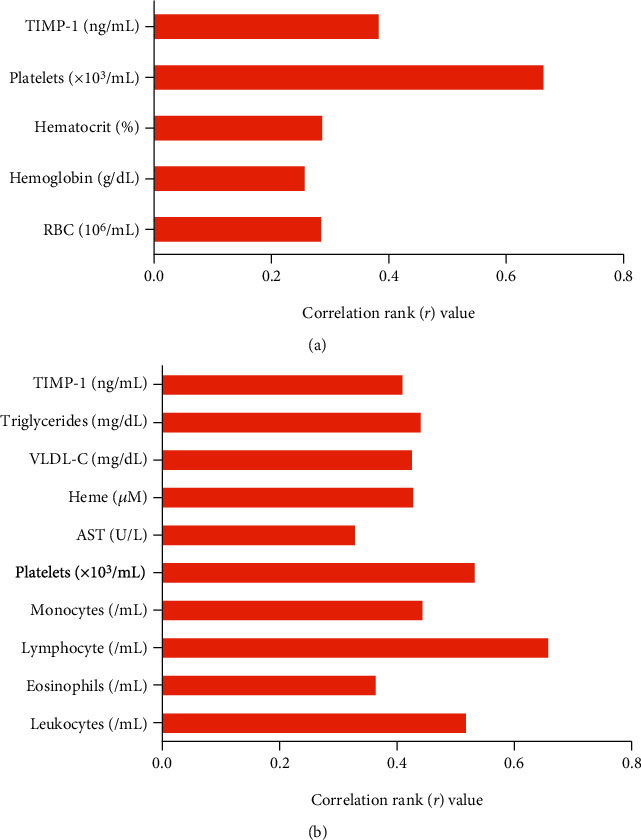
Correlations between TGF-*β*1 levels and laboratory biomarkers in individuals with HbSS and HbSC. (a) Positive correlations between TGF-*β*1 levels and RBC, Hb, Ht, platelet, and TIMP-1 in individuals with HbSS. (b) Positive correlations between TGF-*β*1 levels and leukocytes, eosinophils, lymphocytes, monocytes, platelets, AST, VLDL-C, triglycerides, heme, and TIMP-1 in HbSC individuals.

In individuals with HbSS, TGF-*β*1 was positively correlated with RBC (*r* = 0.282; *p* = 0.0094), Hb (*r* = 0.254; *p* = 0.0197), Ht (*r* = 0.284; *p* < 0.0089), platelets (*r* = 0.663; *p* < 0.0001), and TIMP-1 (*r* = 0.381; *p* = 0.0005) (Figure 2(a)). In addition, in HbSC individuals, TGF-*β*1 was positively correlated with leukocytes (*r* = 0.5168; *p* = 0.0008), eosinophils (*r* = 0.3619; *p* = 0.0236), lymphocytes (*r* = 0.6575; *p* < 0.0001), monocytes (*r* = 0.4421; *p* = 0.0048), platelets (*r* = 0.5318; *p* = 0.0005), AST (*r* = 0.326; *p* = 0.0425), VLDL-C (*r* = 0.424; *p* = 0.0072), triglycerides (*r* = 0.439; *p* = 0.0051), heme (*r* = 0.426; *p* = 0.0076), and TIMP-1 (*r* = 0.408; *p* = 0.0110) (Figure 2(b)).

### 3.4. TGF-*β*1 Plasma Levels Are Associated with Laboratory Parameters in SCD

Considering the fact that no standard or normal clinical range exists with respect to plasma levels of TGF-*β*1 in humans, we endeavored to perform association analyses by creating subgroups of individuals with HbSS and HbSC according to the median values of TGF-*β*1 obtained for each SCD genotype.

**Figure 3:**
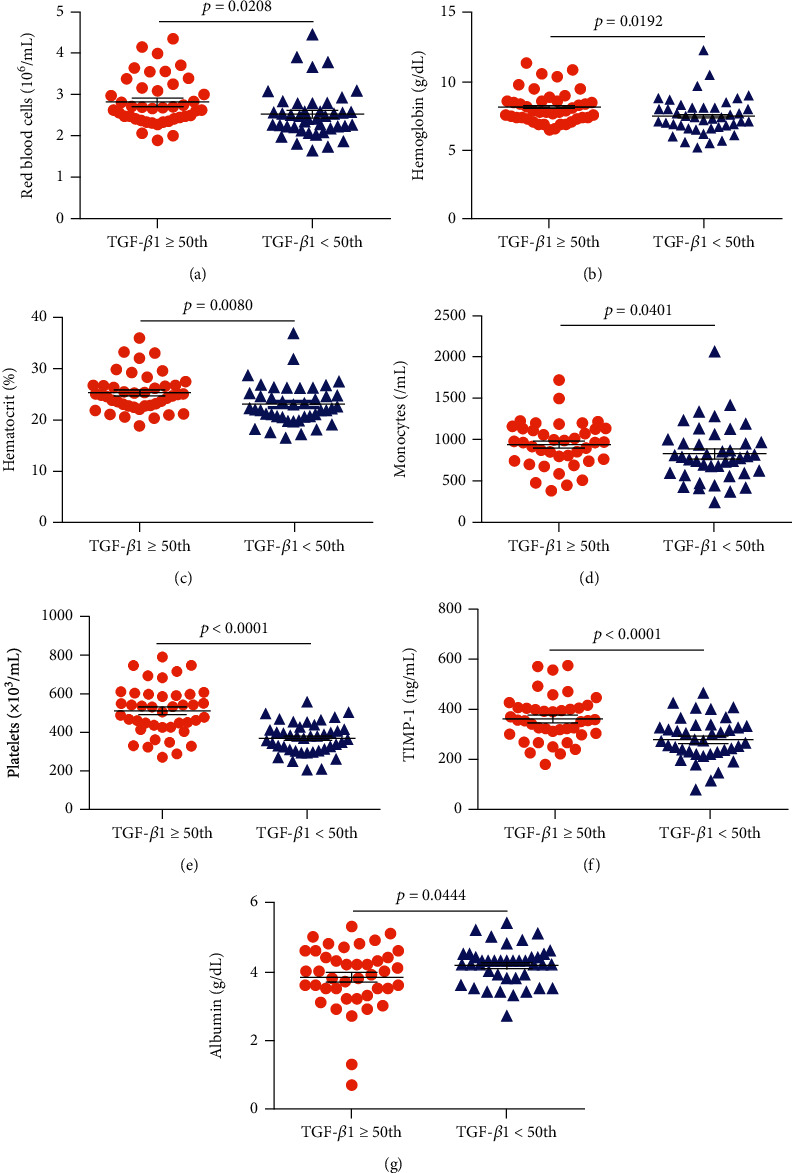
Associations between TGF-*β*1 levels and laboratory biomarkers as well as the glycoprotein TIMP-1 in individuals with HbSS. Individuals with TGF-*β*1 levels above the median (TGF‐*β*1 ≥ 72.29) exhibited increased (a) RBC counts, (b) hemoglobin levels, (c) hematocrit, (d) monocyte counts, (e) platelet counts, and (f) TIMP-1 levels, as well as (g) decreased albumin levels. All *p* values were obtained by the Mann–Whitney *U* test, except for TIMP-1 and platelet counts, for which the independent *t*-test was used.

**Figure 4:**
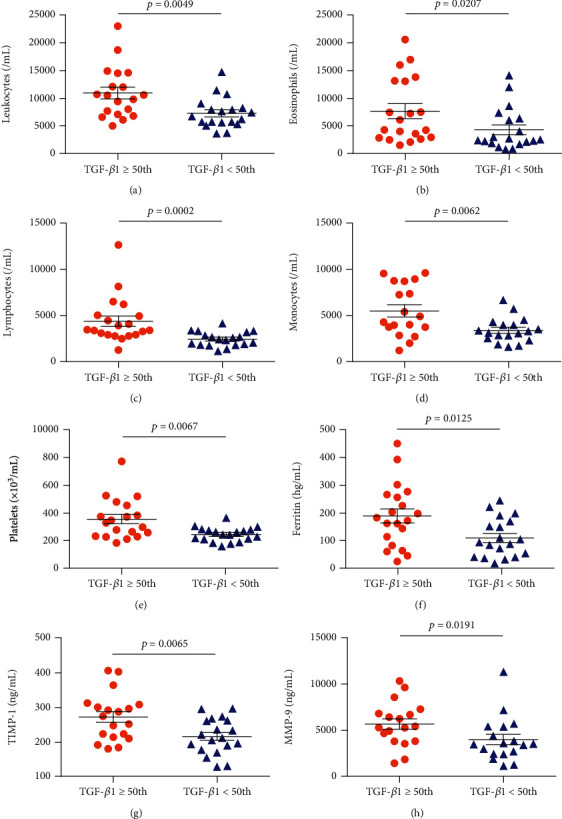
Associations between TGF-*β*1 levels and laboratory biomarkers as well as the glycoprotein TIMP-1 and matrixin MMP-9 in individuals with HbSC. Individuals with TGF-*β*1 levels above the median (TGF‐*β*1 ≥ 47.80) exhibited increased (a) leukocytes, (b) eosinophils, (c) lymphocytes, (d) monocytes, and (e) platelets, as well as higher levels of (f) ferritin, (g) TIMP-1, and (h) MMP-9. All *p* values were obtained by the Mann–Whitney *U* test, except for leukocytes, monocytes, ferritin, and TIMP-1, for which the independent *t*-test was used.

**Figure 5:**
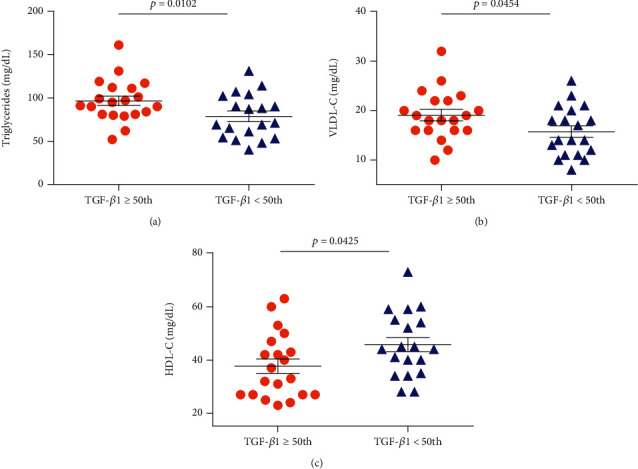
Associations between TGF-*β*1 levels and lipid biomarkers in individuals with HbSC. Individuals with TGF-*β*1 levels above the median (TGF‐*β*1 ≥ 47.80) exhibited (a) increased triglycerides, (b) increased VLDL-C, and decreased (c) HDL-C levels (all *p* values were obtained by the independent *t*-test).

In individuals with HbSS, TGF-*β*1 levels above the median (TGF‐*β*1 ≥ 72.29) were associated with increased RBC (*p* = 0.0208) (Figure 3(a)), hemoglobin (*p* = 0.0192) (Figure 3(b)), hematocrit (*p* = 0.0080) (Figure 3(c)), monocytes (*p* = 0.0401) (Figure 3(d)), platelets (*p* < 0.0001) (Figure 3(e)), and TIMP-1 (*p* < 0.0001) (Figure 3(f)), as well as decreased albumin (*p* = 0.0444) (Figure 3(g)). Among individuals with HbSC, TGF-*β*1 levels above the median (TGF‐*β*1 ≥ 47.80) were associated with increased leukocytes (*p* = 0.0049) (Figure 4(a)), eosinophils (*p* = 0.0207) (Figure 4(b)), lymphocytes (*p* = 0.0002) (Figure 4(c)), monocytes (*p* = 0.0062) (Figure 4(d)), platelets (*p* = 0.0067) (Figure 4(e)), ferritin (*p* = 0.0125) (Figure 4(f)), TIMP-1 (*p* = 0.0065) (Figure 4(g)), MMP-9 (*p* = 0.0191) (Figure 4(h)), triglycerides (*p* = 0.0102) (Figure 5(a)), and VLDL-C (*p* = 0.0454) (Figure 5(b)), as well as decreased HDL-C levels (*p* = 0.0425) (Figure 5(c)).

## 4. Discussion

Despite the fact that several laboratory and genetic biomarkers have been associated with subphenotypes of SCD, in which individuals present a greater propensity of clinical events, the search for prognostic biomarkers of SCD remains challenging [5, 7, 24].

Our analyses showed that HbSS individuals presented increases in biomarkers of hemolysis, leukocytosis, and inflammation, together with decreased levels of HDL-C, in comparison to HbSC and HC individuals. These findings are consistent with previous reports describing HbSS as the most severe form of SCD [25–27]. As individuals with HbSS present more intense hemolysis, anemia tends to be more severe, and hemolytic complications occur more frequently [24, 26].

Herein, higher levels of TGF-*β*1 were found in individuals with HbSS than in those with HbSC and HC. Despite the fact that our case series consisted of pediatric individuals, our results were nonetheless similar to a previous report in steady-state adults with HbSS, HbSC, and HC [17]. The elevated TGF-*β* levels found in HbSS may be associated with endothelial remodeling [12, 28] since the endothelium controls the release of relaxing and contracting factors that regulate localized vascular tone. Vasculopathy, in addition to endothelial dysfunction, are the main chronic events described in SCD and are also involved in the pathogenesis of stroke, renal disease, and pulmonary hypertension [24]. Thus, it is possible that TGF-*β* could be directly involved in the modulation of vasculopathy in SCD individuals. In addition, elevated TIMP-1 levels were detected in individuals with HbSS and HbSC who presented TGF-*β*1 levels above the median. TIMP-1, a protein that modulates cell growth, apoptosis, and angiogenesis, is known to inhibit the catalytic activity of MMP-9 [19]. As TGF-*β*1 stimulates the expression of MMP-2, MMP-9, and TIMP-1, increases in TIMP-1 levels driven by TGF-*β*1 may positively impact angiogenesis [29]. Our results also show that individuals with HbSC who presented TGF-*β*1 levels above the median value also exhibited higher levels of MMP-9, which reinforces the role of TGF-*β* in angiogenesis, vasculopathy, and endothelial dysfunction.

The individuals with HbSS and HbSC who presented with TGF-*β*1 levels above the median also exhibited higher platelet counts than those below the median. This finding is supported by previous studies reporting that more TGF-*β* is produced by platelets than by other cells types; moreover, after activation, platelets rapidly release TGF-*β* [21, 30, 31]. Platelet counts were previously shown to be correlated with TGF-*β* levels in both HbSS and HbSC individuals [17], which corroborate the present correlation analysis.

The positive correlation demonstrated herein between TGF-*β*1 and levels of heme and AST in individuals with HbSC provides evidence of the participation of TGF-*β*1 in hemolysis. Interestingly, genes involved in the TGF-*β*/BMP signaling pathway were previously associated with the clinical manifestations of a hemolytic subphenotype, such as leg ulcers [12].

Our association analysis revealed that individuals with HbSC and TGF-*β*1 levels above the median presented increased leukocyte counts. In addition, our correlation analysis found a positively correlation between TGF-*β*1 levels and leukocyte, eosinophil, lymphocyte, and monocyte counts, indicating the presence of an inflammatory response. Similarly, a previous study reported a correlation between TGF-*β* levels and total leukocyte counts in individuals with HbS*β*-thalassemia [17]. TGF-*β* is involved in neutrophil and monocyte chemotaxis, which influences leukocyte recruitment to inflammatory sites [32]. In the inflammatory state seen in SCD individuals, markedly high expressions of adhesion molecules, as well as the production of chemotactic factors and inflammatory cytokines, all promote leukocyte recruitment [2, 8, 33].

Regarding the lipid profile, HbSC individuals with TGF-*β*1 levels above the median presented decreased HDL-C. In addition, TGF-*β*1 levels were also found to be positively correlated with VLDL-C and triglycerides in these individuals. HDL-C exerts important anti-inflammatory activity in vascular diseases [34–36] In SCD, decreased HDL-C levels have been associated with an inflammatory state [7, 34]. Triglycerides and VLDL-C have been shown to induce inflammatory events in atherosclerosis, supporting the notion that these molecules participate in proinflammatory activity [37].

Our findings suggest that TGF-*β*1 levels bear relations to vasculopathy, endothelial dysfunction, hemolysis, and inflammation in individuals with SCD. Collectively, our results highlight the relevance of investigating novel biomarkers of disease severity in the clinical management of individuals with HbSS and HbSC. To the best of our knowledge, the present study was the first to investigate associations and correlations between TGF-*β*1 levels and hematological and biochemical parameters in a pediatric population with HbSS and HbSC. We emphasize that the identification of markers indicative of a worse prognosis of SCD in children can greatly aid in the improved clinical management of patients.

## 5. Conclusion

The results presented herein suggest that pediatric patients with HbSS present higher levels of TGF-*β*1 than those with HbSC or healthy individuals. The fact that TGF-*β*1 was associated with TIMP-1 in both genotypes indicates that these molecules may play an important role in vascular remodeling and vasculopathy through extracellular matrix deposition. We suggest that since TGF-*β* was found to be associated with hemolysis, leukocytes, platelets, and lipid metabolism, this provides evidence that this immunomarker likely modulates the inflammatory response in SCD.

In summary, the full spectrum of biological effects provoked by TGF-*β* in SCD warrants further investigation, as evidence points to the involvement of this molecule in the pathogenesis of vascular disease.

## Figures and Tables

**Table 1:** Laboratory profile of pediatric patients with sickle cell anemia (HbSS), hemoglobin SC disease (HbSC), and healthy controls (HC).

Parameter	HbSS *N* = 84 (mean ± SD)	HbSC *N* = 39 (mean ± SD)	HC *N* = 59 (mean ± SD)	*p* value
*Hemoglobin pattern*				
Fetal hemoglobin (%)	9.79 ± 6.17	2.69 ± 1.42	0.63 ± 0.51	<0.0001
S hemoglobin (%)	86.64 ± 6.41	51.17 ± 6.31	—	<0.0001^∗^
*Hematological parameters*				
RBC (×10^6^/mL)	2.68 ± 0.59	4.30 ± 0.57	4.70 ± 0.34	<0.0001
Hemoglobin (g/dL)	7.83 ± 1.30	11.14 ± 1.23	12.90 ± 0.94	<0.0001
Hematocrit (%)	24.19 ± 4.00	34.73 ± 3.61	38.79 ± 2.59	<0.0001
MCV (fL)	91.70 ± 10.54	80.67 ± 7.80	82.62 ± 4.77	<0.0001
MCH (*ρ*g)	29.73 ± 3.66	25.89 ± 2.53	27.48 ± 1.79	<0.0001
MCHC (%)	32.39 ± 0.97	32.08 ± 0.94	33.25 ± 0.68	<0.0001
Reticulocyte count (%)	9.25 ± 4.63	4.06 ± 2.50	0.86 ± 0.28	<0.0001
Leukocyte count (/mL)	14734.52 ± 5090.44	9802.56 ± 5988.29	7313.56 ± 2448.04	<0.0001
Neutrophil count (/mL)	5972.00 ± 473.00	4349.00 ± 2095.00	3430.15 ± 1937.21	<0.0001
Eosinophil count (/mL)	751.70 ± 423.40	562.00 ± 462.20	452.36 ± 467.08	<0.0001
Lymphocyte count (/mL)	5953.00 ± 1973.00	3076.00 ± 1241.00	2889.17 ± 957.95	<0.0001
Monocyte count (/mL)	882.00 ± 311.10	459.90 ± 254.80	510.16 ± 221.06	<0.0001
Platelet count (×10^3^/mL)	439.18 ± 128.10	319.33 ± 175.30	313.73 ± 67.59	<0.0001
*Biochemical parameters*				
Total cholesterol (mg/dL)	122.00 ± 25.38	119.00 ± 25.96	157.85 ± 33.41	<0.0001
HDL-C (mg/dL)	32.20 ± 9.40	41.62 ± 12.62	48.19 ± 14.75	<0.0001
LDL-C (mg/dL)	67.39 ± 22.50	59.77 ± 21.03	88.87 ± 33.30	<0.0001
VLDL-C (mg/dL)	22.36 ± 10.01	17.44 ± 5.20	19.60 ± 10.34	0.0180
Triglycerides (mg/dL)	111.96 ± 50.02	87.87 ± 26.16	97.75 ± 51.47	0.0150
Total bilirubin (mg/dL)	3.39 ± 1.81	1.75 ± 1.05	0.57 ± 0.23	<0.0001
Direct bilirubin (mg/dL)	0.81 ± 0.51	0.47 ± 0.27	0.23 ± 0.07	<0.0001
Indirect bilirubin (mg/dL)	2.58 ± 1.66	1.27 ± 0.97	0.30 ± 0.20	<0.0001
LDH (U/L)	1054.20 ± 522.09	518.38 ± 293.41	420.90 ± 87.15	<0.0001
ALT (U/L)	24.31 ± 15.90	20.17 ± 11.78	18.22 ± 8.12	<0.0001
AST (U/L)	56.79 ± 26.92	33.15 ± 14.94	33.78 ± 12.16	<0.0001
Total protein (g/dL)	7.31 ± 0.99	7.24 ± 0.67	7.08 ± 0.65	0.1450
Albumin (g/dL)	3.99 ± 0.77	4.06 ± 0.54	4.19 ± 0.49	0.577
Globulin (g/dL)	3.31 ± 0.86	3.17 ± 0.67	2.89 ± 0.55	0.0080
Ferritin (*η*g/mL)	265.20 ± 209.10	152.20 ± 102.90	31.16 ± 13.57	<0.0001
Urea (mg/dL)	17.55 ± 6.73	19.00 ± 6.49	21.87 ± 6.63	<0.0001
Creatinine (mg/dL)	0.43 ± 0.18	0.54 ± 0.15	0.58 ± 0.18	<0.0001
Alpha-1 antitrypsin (mg/dL)	166.92 ± 40.75	139.95 ± 42.45	148.50 ± 44.04	<0.0001

RBC: red blood cells; MCV: mean corpuscular volume; MCH: mean corpuscular hemoglobin; MCHC: mean corpuscular hemoglobin concentration; HDL-C: high-density lipoprotein cholesterol; LDL-C: low-density lipoprotein cholesterol; VLDL-C: very low-density lipoprotein cholesterol; AST: aspartate aminotransferase; ALT: alanine aminotransferase; LDH: lactate dehydrogenase; *N*: number; SD: standard deviation. All *p* values were obtained using the Kruskal-Wallis test, with the exception of (∗), which indicates a *p* value obtained from the ordinary one-way ANOVA.

## Data Availability

All relevant data used to support the findings of this study are included within the article.
